# The Production of Listeriolysin O and Subsequent Intracellular Infections by *Listeria monocytogenes* Are Regulated by Exogenous Short Chain Fatty Acid Mixtures

**DOI:** 10.3390/toxins12040218

**Published:** 2020-03-30

**Authors:** Erica Rinehart, Julia Chapman, Yvonne Sun

**Affiliations:** Department of Biology, Integrative Science and Engineering Center, University of Dayton, Dayton, OH 45469, USA; erinehart1@udayton.edu (E.R.); jichapman01@gmail.com (J.C.)

**Keywords:** *Listeria monocytogenes*, LLO, short chain fatty acids, *hly*, aerobic, anaerobic

## Abstract

*Listeria monocytogenes* is a foodborne pathogen capable of secreting listeriolysin O (LLO), a pore-forming toxin encoded by the *hly* gene. While the functions of LLO have been studied extensively, how the production of LLO is modulated by the intestinal environment, devoid of oxygen and enriched in short chain fatty acids (SCFAs), is not completely understood. Using *L. monocytogenes* strain 10403s, we found that *hly* transcription was moderately decreased by aerobic SCFA exposures but significantly increased by anaerobic SCFA exposures. Moreover, aerobic, but not anaerobic, exposure to low levels of SCFAs resulted in a significantly higher LLO activity. These results demonstrated that transcriptional and post-transcriptional regulations of LLO production were separately modulated by SCFAs and were responsive to oxygen levels. Examining isogenic mutants revealed that PrfA and SigB play a role in regulating LLO production in response to SCFAs. Effects of SCFAs were also present in the cardiotropic strain 07PF0776 but distinctly different from those in strain 10403s. For both strains, prior exposures to SCFAs altered intracellular infections in Caco-2 and RAW264.7 cells and the plaque sizes in L fibroblasts, a result confirming the ability of *L. monocytogenes* to adapt to SCFAs in ways that impact its subsequent infection outcomes.

## 1. Introduction

*Listeria monocytogenes* is a Gram-positive, facultative anaerobe and a foodborne pathogen. While minor gastrointestinal complications arise in infected immunocompetent individuals, symptoms can be more severe in pregnant women, the elderly, and immunocompromised populations. Approximately 1600 people annually are estimated to become infected with *L. monocytogenes* and hospitalized in the United States, among which as many as one in five patients do not survive, making *L. monocytogenes* infections contributing to 19% of the overall deaths related to foodborne pathogens [1]. As a result of the high mortality rate, *L. monocytogenes* is under stringent surveillance in the food industry and is responsible for $2.8 billion of costs associated with recalls and medical expenses annually [2]. 

Transmission and infection of *L. monocytogenes* rely on multiple virulence genes contributing to various stages of pathogenesis—surviving and colonizing the host gastrointestinal tract, binding to and entering host cells, growing inside the host cell cytosol, and spreading from one host cell to another. Therefore, to coordinate appropriate expression of virulence genes, *L. monocytogenes* needs to be well in-tune with its environmental cues. For example, transcription of the *hly* gene, which encodes listeriolysin O (LLO)—a cholesterol-dependent pore forming toxin that facilitates *L. monocytogenes* entry into the host cell cytosol [3], is responsive to a multitude of environmental factors, ranging from salt concentrations [4], growth temperature [5], to culture pH [5,6]. We have also shown that anaerobic conditions and propionate, two important environmental signals present in the intestinal lumen, can impact LLO production [7,8,9]. 

Propionate is one of the common intestinal short chain fatty acids (SCFAs), which are byproducts of bacterial fermentation of non-digestible carbohydrates and dietary fibers in the gut [10,11]. Fermentation of carbohydrates by the endogenous microbes in the gastrointestinal lumen can yield 500–600 mmol of SCFAs daily, most of which are absorbed and serve as an energy source for the host, accounting for 5–15% of human energy requirements [12,13]. SCFAs also regulate colonic pH, colonic epithelial cell volume, and a variety of cellular functions, including proliferation, differentiation, and gene expression [14,15,16,17,18]. Moreover, SCFAs are important modulators of leukocyte function, such as chemotaxis, oxidative burst, phagocytosis, and microbial killing [19,20,21,22]. Therefore, individual variations in SCFA levels may underline important clinical implications and pathogenesis [23]. However, very little is known whether the level of SCFAs plays a role in individual susceptibility to *L. monocytogenes* infections. 

Butyrate, one of the major SCFAs found in the human intestines along with acetate and propionate, exhibits a strong inhibitory activity on LLO production by *L. monocytogenes*, an activity likely mediated by the loss of branched chain fatty acids (BCFAs) [8]. The membrane fatty acid composition of *L. monocytogenes*, when grown under standard laboratory conditions, is made up of approximately 90% anteiso-BCFAs, specifically anteiso-C_15:0_ and C_17:0_ [8,24,25]. The synthesis of BCFAs is catalyzed mainly by the branched chain alpha-keto acid dehydrogenase complex (BKD), a lipoic acid-dependent enzyme complex responsible in forming the branched chain amino acid-derived substrates for BCFA synthesis [26]. Transposon insertion mutants of BKD have dramatically decreased levels of BCFAs (<40% of WT) and exhibited severely compromised fitness phenotypes and LLO production [27,28], similarly to butyrate-supplemented wildtype bacteria. Together, these reports highlighted the role of BCFAs in *L. monocytogenes* fitness and pathogenesis and further suggest a potential effect of butyrate on *L. monocytogenes* during the intestinal phase of infection. However, all the earlier studies were performed under aerobic conditions, which are relevant conditions during *L. monocytogenes* intracellular infection but are not representative conditions during *L. monocytogenes* exposure to SCFAs in the anaerobic lumen of the intestines. 

In a subsequent study investigating the effects of propionate on *L. monocytogenes* under aerobic as well as anaerobic conditions identified that the production of LLO was highly sensitive to regulation by exogenous propionate [9]. More specifically, while aerobic propionate exposure resulted in a dose-dependent decrease in LLO production, anaerobic propionate exposure resulted in a dose-dependent increase in LLO production [9]. Given that overall BCFA levels were also decreased in propionate-treated *L. monocytogenes* under aerobic as well as anaerobic conditions [9], it is clear that the general loss of BCFAs upon propionate or butyrate exposure is not the only mechanism mediating the regulation of LLO production. Moreover, these results highlight a potential opposing effect of individual SCFAs on LLO production and justify a need to consider the effects of SCFAs on *L. monocytogenes* as mixtures in addition to individual components to identify potential interactions. 

In this study we investigated the effects of exposure to SCFA mixtures, at two different combinations (M_lo_ and M_hi_) under aerobic or anaerobic conditions, on growth, membrane fatty acid composition, LLO production, and subsequent infections to gain a better insight into *L. monocytogenes* behavior in response to SCFAs during intestinal transit. The SCFA mixtures were used to obtain a better understanding of the effects of SCFAs as one integrated, physiologically relevant signal. We also examined the effects of SCFAs on transcription factor mutants with constitutively active PrfA or deletion in *sigB* to further investigate the regulatory mechanism underlying *L. monocytogenes* response to SCFAs. Finally, we compared the effects of SCFAs on LLO production as well as subsequent infections in two different strains, 10403s and 07PF0776, to determine the relevance of our observations. We found that, compared to no SCFA controls, supplementation of SCFAs resulted in distinctive changes in the transcriptional and post-transcriptional regulations of LLO production, altering subsequent infection outcomes in a manner sensitive to oxygen levels. These results highlight the ability of *L. monocytogenes* to detect and respond to SCFAs and suggest the SCFA levels in the intestine might play a role in determining the outcome of *L. monocytogenes* infections.

## 2. Results

### 2.1. High but not Low Levels of SCFAs Reduced In Vitro Growth

The growth of *L. monocytogenes* under these conditions was evaluated by culture optical density and culture pH measurements. Compared to non-supplemented controls, SCFA supplementations at M_lo_ or M_hi_ levels both resulted in a significantly higher culture pH but only supplementation at the M_hi_ level resulted in a significantly decreased culture optical density under aerobic or anaerobic conditions (Table 1). To ensure the effects of SCFA mixtures on growth was not a result of higher salt levels, we also tested the effects of salt on *L. monocytogenes* growth and found that supplementations of NaCl at levels as high as 200 mM did not significantly affect *L. monocytogenes* growth (data not shown). Doubling times were also calculated from growth curves, showing that supplementations of SCFAs at higher (M_hi_) but not lower (M_lo_) levels resulted in a significantly higher doubling time when compared to no supplementation controls (Table 1). Similar effects of SCFAs on doubling time were also observed in isogenic mutants (Table 1) with a constitutively active virulence regulator PrfA (PrfA*) or a deletion in the stress response sigma factor gene *sigB* (Δ*sigB*). These results confirmed that higher levels of SCFAs (M_hi_) reduced but did not completely inhibit in vitro growth under aerobic or anaerobic conditions. 

To determine whether the effects of SCFA supplementations on growth were related to pH variations, we measured overnight culture density and pH in phosphate buffered BHI (Table 2). In the absence of SCFAs, pH optimal for growth in buffered BHI was notably different between aerobic and anaerobic cultures. Whereas BHI buffered at pH = 6.0 yielded the highest culture optical density under aerobic conditions, BHI buffered at pH = 7.0 yielded the highest culture optical density. The addition of SCFAs in general resulted in decrease in growth but the extent of the effects varied among media buffered at different pH values. For example, in BHI buffered at pH = 6.0, addition of M_hi_ levels of SCFAs resulted in growth inhibition that was not observed in media without buffering or media buffered at PH = 7.0 or 8.0. Significant changes in culture pH by SCFA supplementations were also observed when compared to no SCFA controls under the same oxygen levels. In general, a decrease in growth corresponds to increase in culture pH, but growth decrease was also observed in anaerobic, M_hi_ SCFA-treated cultures where culture pH was not significantly changed. Therefore, the effects on growth by SCFAs are not mediated exclusively by pH modifications.

### 2.2. SCFA Supplementations Altered Membrane Fatty Acid Compositions

**Figure 1:**
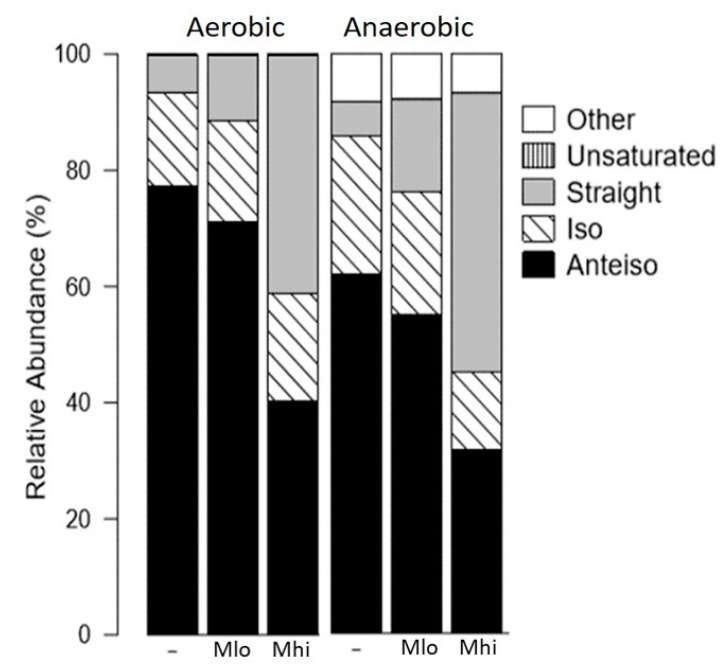
Fatty acid composition (in percentages) varies in *L. monocytogenes* strain 10403s grown in BHI supplemented with or without M_lo_ or M_hi_ SCFA mixtures. Averages of 2 independent replicates were plotted. “Straight” indicates straight chain fatty acids. “Iso” and “Anteiso” indicate iso- and anteiso-branched chain fatty acids. Details of specific fatty acids are listed in Table 3 and Table 4.

Both propionate and butyrate, separately, have been demonstrated to decrease the abundance of branched chain fatty acids (BCFAs) and increase the abundance of straight chain fatty acids in *L. monocytogenes* [8,9]. Here we investigated how the membrane fatty acid composition was affected by supplementation of SCFAs in mixtures and whether there was an oxygen-dependent alteration of fatty acid composition in response to SCFA exposure. *L. monocytogenes* from overnight cultures, grown aerobically or anaerobically with or without SCFA mixtures, were collected, frozen, and sent for FAME analysis by Microbial ID (Newark, DE). In general, BCFAs represented the most abundant fatty acids in untreated samples (Figure 1, Table 3 and Table 4) with the production of modified fatty acids only present in anaerobically grown *L. monocytogenes* (Table 4). Moreover, the proportions of straight chain fatty acids were increased by SCFA supplementations under aerobic or anaerobic conditions (Figure 1). More specifically under aerobic conditions (Table 3), the proportion of total BCFAs was reduced to 58.5% in *L. monocytogenes* treated with M_hi_ SCFAs, compared to 93.42% for bacteria without SCFA supplementations or 88.41% for bacteria with M_lo_ SCFA supplementations. Similarly, under anaerobic conditions (Table 4), the proportion of total BCFAs in cultures supplemented with SCFAs at the M_hi_ level samples was reduced to 45.2%, compared to 85.93% for no supplementation or 76.1% for M_lo_-supplemented samples. 

While the reduction of total BCFAs was observed in both aerobic and anaerobic conditions, the specific changes in different BCFAs appeared to respond to the presence or absence of oxygen. Under aerobic conditions, the loss of BCFAs was attributed entirely to the loss of anteiso-BCFAs, decreasing from 77.39% in control samples to 71.11% and 40.41% in cultures supplemented with SCFAs at the M_lo_ or M_hi_ levels, respectively (Table 3). The proportion of odd number iso-BCFAs exhibited a minimal, if any, decrease while the proportion of even number iso-BCFAs exhibited a small increase in both M_lo_- and M_hi_-supplemented samples (Table 3). Under anaerobic conditions, in contrast, the loss of total BCFAs was attributed to the loss of anteiso-BCFAs as well as odd number iso-BCFAs (Table 4). The proportion of even number iso-BCFAs exhibited a small increase in M_lo_-supplemented samples but a decrease in M_hi_-supplemented samples (Table 4). 

Similarly, the accompanying general increase in the proportion of total straight chain fatty acids in response to SCFA supplementations was observed under both aerobic and anaerobic conditions but with distinct differences in the subtype composition. Supplementations with SCFAs at the M_hi_ level resulted in the most increase in total straight chain fatty acids, from 6.655% to 41.085% under aerobic conditions and 5.85% to 48.11% under anaerobic conditions (Table 3 and Table 4). However, the enrichment of straight chain fatty acids was more apparent in anaerobic samples than aerobic samples. Under aerobic conditions, supplementations of M_lo_ and M_hi_ SCFA mixtures resulted in a 1.73- and 6.17-fold increase in total straight chain fatty acids, respectively. Under anaerobic conditions, in contrast, supplementation with M_lo_ and M_hi_ SCFA mixtures resulted in a 2.75- and 8.22-fold increase in total straight chain fatty acids, respectively. These results indicate that *L. monocytogenes* contains the ability to utilize SCFAs and as a result modifies its membrane composition. Also, substrate utilization for fatty acid synthesis is likely modulated by oxygen.

### 2.3. Anaerobic Transcription of Hly was Enhanced by SCFAs

**Figure 2:**
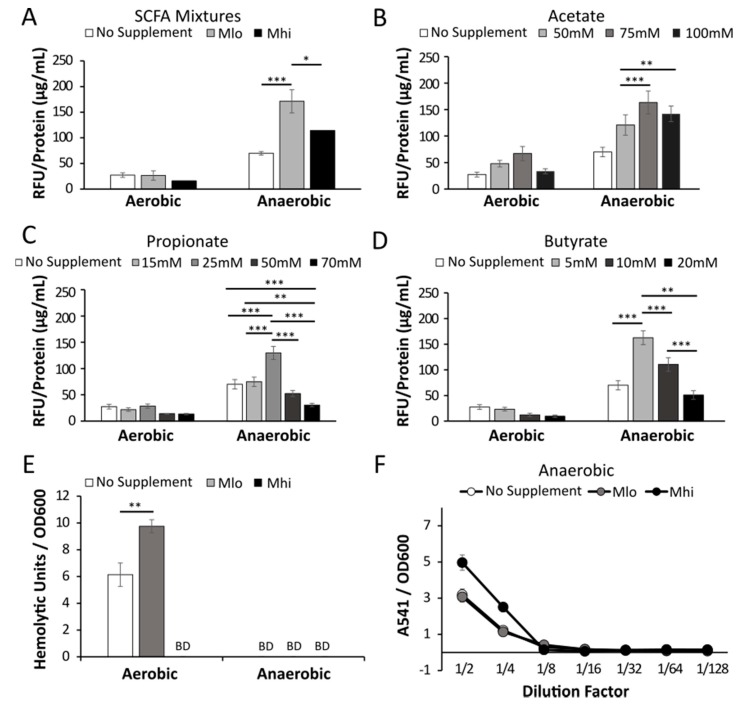
Supplementations of SCFAs alter *hly* transcription levels and supernatant LLO activity. Using an *hly* transcriptional reporter, relative fluorescence units (RFU) were obtained from GUS reporter assays and normalized to sample protein concentrations (µg/mL) to assess the level of *hly* transcription in bacteria grown in BHI supplemented with or without SCFA mixtures (**A**), acetate (**B**), propionate (**C**), or butyrate (**D**). Averages of 9 replicates from three independent experiments were plotted with error bars representing SEM. Significant differences between samples are indicated by: * *p* < 0.05, ** *p* < 0.01, *** *p* < 0.001. The impact of aerobic or anaerobic SCFA supplementations on supernatant listeriolysin O (LLO) activity (**E**,**F**). “BD” represents values below detection or a level lower than half complete lysis, thus unable to calculate hemolytic units. For anaerobic cultures, hemolytic assay absorbance values, normalized by culture optical densities, were shown to highlight differences among samples (F). Averages of three replicates were plotted with error measurements indicating SD. Results were representative of at least three independent experiments. Significant difference between samples are indicated by: * *p* < 0.05, ** *p* < 0.01, *** *p* < 0.001.

The effects of SCFAs on *L. monocytogenes* listeriolysin O (LLO) production were tested at the transcriptional level using a *hly* transcriptional reporter strain. The reporter strain was cultured overnight in BHI supplemented with low (M_lo_) or high (M_hi_) SCFA mixtures [29] (Figure 2A) or individual concentrations of SCFAs in ranges encompassing those in the SCFA mixtures to isolate the contributing SCFA and determine potential additive effects of these SCFAs (Figure 2B,C). Under aerobic conditions, supplementations of SCFA mixtures resulted a notable but not significant decrease in *hly* transcription (Figure 2A). In contrast, under anaerobic conditions, SCFA supplementations at the low M_lo_ levels resulted in a significantly elevated *hly* transcription (Figure 2A). Effects of individual SCFA supplementation resulted in a similar pattern. Under aerobic conditions, after averaging 3 independent experiments each performed with triplicates, supplementations of acetate, propionate, or butyrate individually did not significantly alter *hly* transcription, although an increasing trend with acetate and a decreasing trend with propionate and butyrate were noted (Figure 2B–D). Under anaerobic conditions, supplementations of individual SCFAs resulted in a significantly elevated *hly* transcription (Figure 2B–D). However, the relationship between concentrations of individual SCFAs and *hly* transcription levels was not linear. The enhancing effects of individual SCFAs under anaerobic conditions peaked at 75 mM for acetate, 25 mM for propionate, and 5 mM for butyrate over the tested range. 

To assess potential post-transcriptional effects of SCFAs on LLO production, we measured the activity of secreted LLO in culture supernatant as a proxy for the overall LLO production. Under aerobic condition, supplementations with M_lo_ SCFAs resulted in a significant increase in LLO activity while supplementations with M_hi_ SCFAs resulted in a compromised LLO activity (Figure 2E). Because LLO activity in anaerobically grown cultures was too low to calculate hemolytic activity, we also showed the results in a dilution curve and noted an elevated LLO activity in cultures supplemented with M_hi_ SCFAs (Figure 2F). These results together indicate that the activity of LLO by *L. monocytogenes* can be enhanced after exposure to SCFAs with the effects highly dependent on the presence of absence of oxygen. 

**Figure 3:**
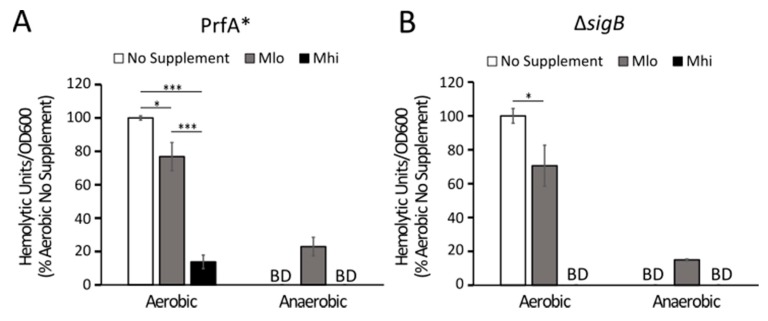
Supplementations of SCFAs alter supernatant LLO activity in the PrfA* (**A**) and Δ*sigB* (**B**) mutants of strain 10403s. The impact of aerobic or anaerobic SCFA supplementations on supernatant LLO activity was determined in overnight cultures. Hemolytic units were normalized by overnight culture optical densities and then compared to the no supplementation control in each strain. “BD” represents values below detection or a level lower than half complete lysis, where hemolytic units could not be calculated. Averages of three independent experiments (n = 9) were plotted with error measurements indicating SEM. Significant difference between samples are indicated by: * *p* < 0.05, ** *p* < 0.01, *** *p* < 0.001.

To explore potential mechanisms that result in altered LLO production in response to SCFAs, we examined the effects of SCFAs on LLO production in isogenic mutants with constitutive active PrfA (PrfA*) or deficient SigB (Δ*sigB*). Both PrfA and SigB are known transcriptional regulators that can influence LLO production [30] but their roles in *L. monocytogenes* sensing SCFAs have not been investigated prior to this study. Under aerobic conditions, supplementation of SCFAs resulted in a significant, dose-dependent decrease of LLO activity in both the PrfA* and the Δ*sigB* mutants (Figure 3). This was in direct contrast to the enhancing effect of aerobic M_lo_ SCFA exposure on the wildtype bacteria (Figure 2E). Under anaerobic conditions, supplementations of M_lo_, not M_hi_, resulted in a significant increase in supernatant LLO activity. These results indicate that while the regulation of LLO production in these mutants remain sensitive to perturbations by SCFAs under aerobic or anaerobic conditions, the sensitivity to different levels of SCFAs appears to be altered in the presence of constitutively active PrfA or in the absence of SigB.

### 2.4. Production of LLO in Strain 07PF0776 Was Sensitive to Regulation by SCFAs 

**Figure 4:**
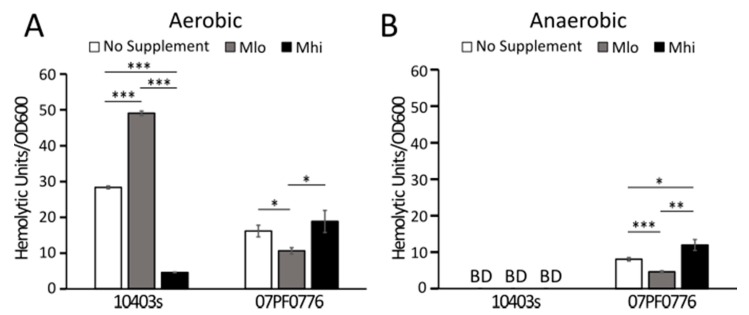
Supplementations of SCFA mixtures alter supernatant LLO activity in strains 10403s and 07PF0776. The impact of aerobic (**A**) or anaerobic (**B**) SCFA supplementations on supernatant LLO activity was determined in both strain 10403s and the cardiotropic strain 07PF0776. Hemolytic units were normalized by culture optical densities. “BD” represents values below detection or a level lower than half complete lysis, where hemolytic units could not be calculated. Averages of three replicates were plotted with error measurements indicating SD. * *p* < 0.05, ** *p* < 0.01, *** *p* < 0.001. BD signifies below detection. Results represent 3 independent experiments.

To establish the relevance of our observations in strain 10403s, we also investigated the effects of SCFA exposure on LLO production in the cardiotropic strain 07PF0776 (serotype 4b) [31] to identify any strain-dependent responses to SCFAs. Strain 07PF0776 in general exhibited lower supernatant LLO activities under aerobic conditions (Figure 4A) but higher LLO activities under anaerobic conditions (Figure 4B) than strain 10403s. There were also distinctive differences in responses to SCFA mixtures. Under aerobic conditions (Figure 4A), while supplementations of M_lo_ SCFAs resulted in a significantly increased LLO activity in strain 10403s, they resulted in a significantly decreased LLO activity in strain 07PF0776. Under anaerobic conditions, supplementations of M_lo_ SCFAs resulted in a significant increase in LLO activity in strain 07PF0776 (Figure 4B), similarly to strain 10403s (Figure 2F). However, anaerobic supplementations of M_lo_ SCFAs resulted in a significant decrease in LLO activity in strain 07PF0776 (Figure 4B), compared to the lack of effect of M_lo_ SCFAs in strain 10403s (Figure 2E). These results indicate that while the effects of SCFAs on strain 10403s are more sensitive to perturbations by the presence or absence of oxygen than those on strain 07PF0776, both strains are capable of sensing and responding to SCFAs by altering LLO production.

### 2.5. Prior SCFA Exposure Altered Subsequent Infection Outcomes in Tissue Culture Infections

The regulatory effects of SCFAs on LLO production suggest a potential impact on subsequent intracellular infections after SCFA exposure. Therefore, we infected Caco-2 intestinal epithelial cells and RAW264.7 macrophages with *L. monocytogenes* grown with or without supplementations of M_lo_ or M_hi_ SCFA mixtures under aerobic or anaerobic conditions and assessed subsequent intracellular infections in a standard gentamicin protection assay. It is important to point out that in these experiments, the host cells were never exposed to exogenous SCFAs prior to infection. SCFAs were never added to infected cells throughout infections. Any effects from SCFA exposure were the result of *L. monocytogenes* adaptations to SCFAs and/or the host responses to differentially adapted *L. monocytogenes*. 

**Figure 5:**
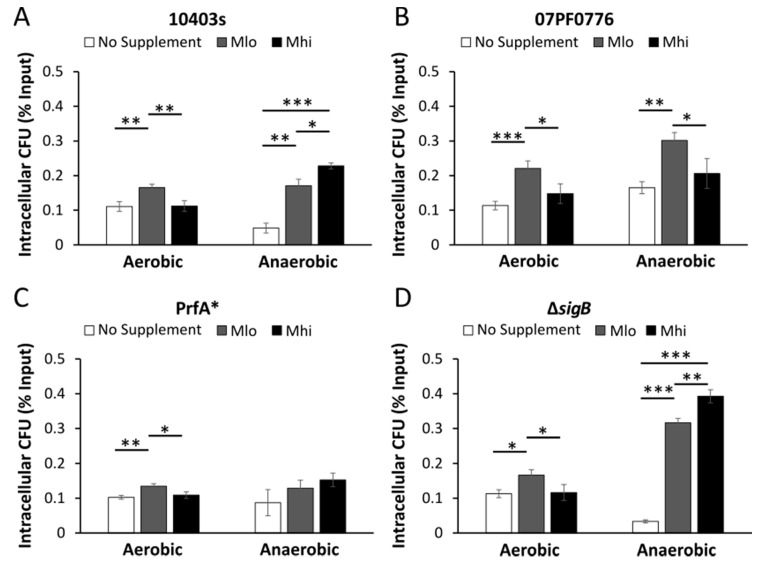
Supplementations of SCFA mixtures alter *L. monocytogenes* invasion in non-polarized Caco-2 intestinal epithelial. The impact of aerobic or anaerobic pre-exposure to SCFAs on *L. monocytogenes* epithelial cell invasion was determined for strain 10403s (**A**), the cardiotropic strain 07PF0776 (**B**), and the mutants PrfA* (**C**) and Δ*sigB* (**D**). PrfA* and Δ*sigB* are isogenic strains of wildtype strain 10403s. Cells were infected with a MOI of 10 and lysed at 2 hpi to determine *L. monocytogenes* invasion. Averages of three replicates were plotted with error measurements indicating SD. * *p* < 0.05, ** *p* < 0.01, *** *p* < 0.001. BD signifies below detection. Results represent 2 independent experiments.

**Figure 6:**
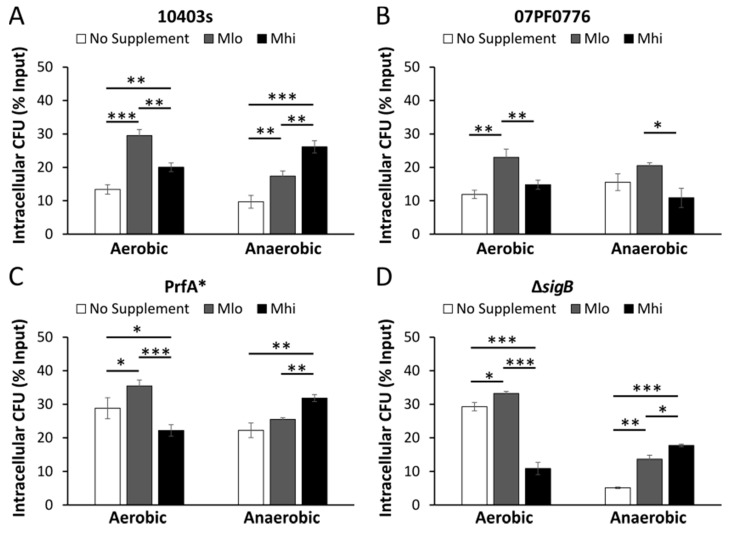
Supplementations of SCFA mixtures alter *L. monocytogenes* intracellular survival in RAW264.7 macrophages. The impact of aerobic or anaerobic pre-exposure to SCFAs on *L. monocytogenes* intracellular survival in macrophages was determined for strain 10403s (**A**), the cardiotropic strain 07PF0776 (**B**), and the mutants PrfA* (**C**) and Δ*sigB* (**D**). PrfA* and Δ*sigB* are isogenic strains of wildtype strain 10403s. Cells were infected with a MOI of 10 and lysed at 2 hpi to determine *L. monocytogenes* early intracellular survival. Averages of three replicates were plotted with error measurements indicating SD. * *p* < 0.05, ** *p* < 0.01, *** *p* < 0.001. BD signifies below detection. Results represent 3 independent experiments.

In non-polarized Caco-2 cells infected by aerobically grown bacteria, for all four strains tested, prior bacterial exposure to M_lo_ SCFA mixtures resulted in a significant increase in the percentages of intracellular bacteria (Figure 5), an observation indicative of an enhancement of invasion by aerobic exposure to low levels of SCFA mixtures regardless of genotype. In contrast, in non-polarized Caco-2 cells infected by anaerobically grown bacteria, supplementation of SCFAs in general showed a dose-dependent increase in the percentages of intracellular WT and isogenic mutants of strain 10403s but not strain 07PF0776 (Figure 5). Anaerobic exposure to high levels of SCFA mixtures significantly enhanced invasion by strain 10403s but not strain 07PF0776. Similar phenotypes were also observed in the infected RAW264.7 macrophages (Figure 6) where the effects of SCFA exposure on subsequent infections were similar among WT and isogenic mutants of strain 10403s (Figure 6A,C,D). Also, the effects of anaerobic, but not aerobic, exposure to high levels of SCFA mixtures on subsequent infections were distinctively different between strains 10403s and 07PF0776 (Figure 6A,B). 

**Figure 7:**
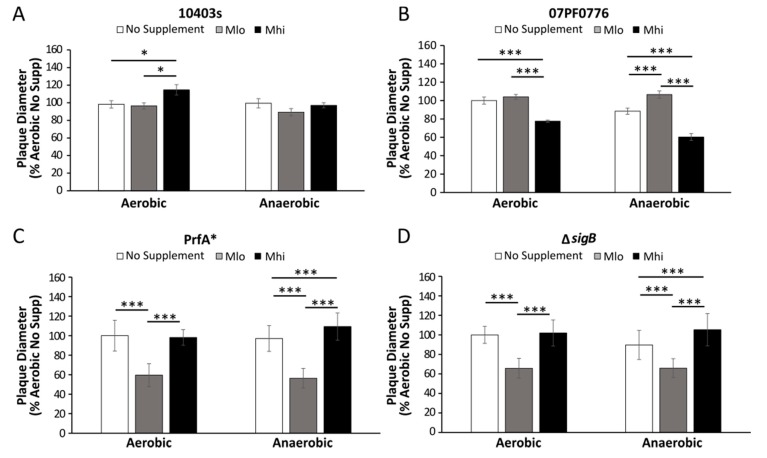
SCFA pre-exposures by *L. monocytogenes* prior to infections alter plaque sizes in infected L fibroblasts. The impact of aerobic or anaerobic SCFA pre-exposures by *L. monocytogenes* on plaque sizes in subsequent infections in L-cell monolayers was determined for both strains 10403s (**A**) and 07PF0776 (**B**) and 10430s isogenic mutants PrfA* (**C**) and Δ*sigB* (**D**). Plaques were visualized two days post infection. Percent plaque sizes were calculated based on comparisons to the average plaque sizes by cells infected by bacteria without SCFA pre-exposures. Averages of 30 plaques were plotted with error measurements indicating SD. Results represent 3 independent experiments. * *p* < 0.05, ** *p* < 0.01, *** *p* < 0.001.

To further investigate the consequences of SCFA exposure on *L. monocytogenes* pathogenesis, an L-cell plaque assay was performed with strains 10403s and 07PF0776 to evaluate the effects of SCFA exposure and adaptation on subsequent infection outcomes. Overnight cultures of *L. monocytogenes* grown with or without SCFAs under aerobic or anaerobic conditions were washed to remove any residual SCFAs and used to infect monolayers of L fibroblast cells. It is important to point out that SCFAs were not included throughout the infection from seeding the cells to measuring plaque sizes at 2 days post infections. Therefore, plaque sizes represent the effects of prior SCFA exposure on the ability of *L. monocytogenes* to establish an intracellular life cycle in subsequent infections. For strain 10403s, aerobic pre-exposures to M_hi_ SCFA mixture, but not other conditions tested, resulted in a significant increase in plaque diameters (Figure 7A). However, this was not seen in plaques created by the isogenic mutants (Figure 7C,D), indicating that *normal* PrfA and SigB activities may play a role in cell-to-cell spread in adaptations to M_lo_ SCFAs and oxygen exposure that are relevant for long-term infection success. In contrast, aerobic pre-exposures to M_hi_ SCFAs by strain 07PF0776 resulted in a significant decrease in plaque diameters (Figure 7B). In cells infected by anaerobically grown strain 07PF0776, pre-exposures to M_lo_ SCFA mixture prior to infections resulted in a significant increase in plaque sizes compared to no SCFA controls (Figure 7B). However, pre-exposures to M_hi_ SCFA mixture prior to infections resulted in a significant decrease in plaque sizes compared to no SCFA controls (Figure 7B). These results confirm the presence of strain variations in *L. monocytogenes* response to SCFAs and suggest the possibility of SCFAs in the intestinal lumen influencing *L. monocytogenes* infection outcome in a strain-dependent manner.

## 3. Discussion

In this study we reported that membrane fatty acid composition and production of LLO were sensitive to regulation by SCFA mixtures in a manner that was modulated by oxygen availability in *L. monocytogenes* strain 10403s. The response to SCFAs was not exclusive to strain 10403s because the effects of SCFAs on LLO production were also observed in strain 07PF0776 with distinct differences. Most notably, pre-exposures to SCFAs prior to infections resulted in a sustained effect on plaque sizes two days post infection despite the lack of SCFAs throughout the infection. By using SCFA mixtures as an integrated signal, these results together suggest for the first time that the intestinal environment, such as levels of SCFAs and oxygen availability, may play a key role in determining strain variations in virulence or the individual susceptibility to *L. monocytogenes* infections. 

### 3.1. SCFA Metabolism and Fatty Acid Synthesis

SCFAs tested in this study are structurally related compounds differing only by the length of the carbon chain. However, *L. monocytogenes* is capable of distinguishing between these three SCFAs—with the highest sensitivity to butyrate, followed by propionate and acetate, and responding to these SCFAs by altering *hly* transcription and LLO production. To begin to dissect the likely complex regulatory mechanism, we explored SCFA metabolism as a potential component in mediating the effects of SCFAs on the regulation of LLO production. The pathways for SCFA metabolism all converge at fatty acid synthesis. While acetate may be directly incorporated into the central metabolism through acetyl-CoA, butyrate and propionate are likely being converted to butyryl- and propionyl-CoA, respectively, first through phosphorylation by kinases (LMRG_00611 and LMRG_00820), followed by the activity of phosphotransbutyrylase (LMRG_00819) that catalyzes the transfer of C3-C5 acyl chain to CoA molecules [32], forming precursors for the synthesis of straight chain fatty acids. As a result, exposure to SCFAs resulted in a dose-dependent increase in straight chain fatty acids (Table 3 and Table 4). 

Membrane fatty acid composition is highly sensitive to fluctuations in environmental conditions such as temperature [33] and can influence important biological functions. *L. monocytogenes* membrane fatty acids are dominated by BCFAs, including anteiso-, odd iso-, and even-iso BCFAs. Growth at temperatures below 10 °C results in an enrichment of anteiso-C_15:0_ branched chain fatty acids (BCFAs) [24,28,34,35,36,37,38], contributing to homeoviscous adaptation to low temperatures [34,39]. Response and adaptation to pH [25] or adherent growth [40] have also been reported to involve modifications in the fatty acid composition. We showed here that oxygen levels similarly served as an environmental determinant in altering fatty acid composition. While the decreased level of total branched chain fatty acids after SCFA exposure was observed under aerobic and anaerobic conditions, anaerobic modification of membrane fatty acid by SCFAs encompasses a decrease in anteiso-BCFA but an increase in iso-BCFAs, resulting in an overall decrease in the ratio of anteiso- to iso-BCFA of 2.6, a much lower value than the ratio of 4.8 observed under aerobic conditions. With anteiso-BCFAs conferring a higher degree of membrane fluidity than iso-BCFAs [26], it is likely that anaerobically grown *L. monocytogenes* exhibits a reduced membrane fluidity in comparison to aerobically grown bacteria upon exposure to SCFAs despite the similar growth temperature (37 °C). The extent by which SCFA exposure alters membrane fluidity under aerobic versus anaerobic conditions remains to be experimentally determined. Nevertheless, a reduction in membrane fluidity can render *L. monocytogenes* more susceptible to antimicrobial peptides [8] and suggests that reducing oxygen levels, such as modified atmosphere packaging [41], might be key in compromising *L. monocytogenes* fitness and environmental transmission. Moreover, although SCFA supplementations resulted in a dramatic change in *L. monocytogenes* fatty acid composition, supporting the utilization of SCFAs as substrates for fatty acid synthesis, whether the metabolism of these SCFAs is required for regulating LLO production remains to be determined. 

### 3.2. Transcriptional and Post-Transcriptional Regulations of LLO Production by SCFAs

The transcriptional regulation of LLO production has been extensively investigated, establishing PrfA and SigB as two major transcription factors that can influence LLO production. PrfA is the main transcription activator for many virulence genes in *L. monocytogenes* [42,43,44,45] while SigB is a stress response sigma factor [46] that can also influence *prfA* expression [47,48,49,50]. Because the extremely low level of LLO production in a PrfA-deficient mutant, we utilized a mutant with constitutively activated PrfA [51] to investigate the role of PrfA in regulating LLO production in response to SCFAs. Previous work from our lab has demonstrated that in both PrfA* and Δ*sigB* mutants, LLO activities in anaerobic culture supernatants remained significantly lower than those in aerobic culture supernatants [7], a result supporting that the compromised LLO protein production takes place independently of the normal PrfA or SigB activities. In this study, both PrfA* and Δ*sigB* mutants, regardless of oxygen levels, exhibited an altered response to low, but not high, levels of SCFA mixtures (Figure 4). These observations are indicative of PrfA and SigB contributing, at least partially, to the regulation of LLO production in response to SCFAs. Moreover, they strongly suggest that the effects of SCFAs on LLO production are not linear and are likely mediated by different mechanisms at low versus high levels. 

Our results also reveal that these environmentally important signal—oxygen availability and SCFA levels, exert a separate and often opposing effect on the transcriptional and post-transcriptional regulations of LLO production in strain 10403s. Under anaerobic conditions where LLO activity was low (Figure 2E,F), the *hly* transcription level remained high (Figure 2A–D). The lack of correlation between transcription and protein activity suggests that while anaerobicity in general results in higher *hly* transcription, LLO protein production is dependent on the presence of oxygen. In other words, the anaerobic signal exerts an opposing effect on the transcriptional and post-transcriptional regulation of LLO production. In the presence of low levels of SCFAs under aerobic conditions, the lack of transcriptional response (Figure 2A) surprisingly led to a significantly higher supernatant activity (Figure 2E). In contrast, low levels of SCFAs under anaerobic conditions induced a strong transcriptional upregulation without the corresponding increase in supernatant activities. These results confirm that LLO protein production is likely an oxygen dependent process or there exists a strong inhibitory signal on LLO protein production under anaerobic conditions. The specific underlying mechanisms remain to be determined but may reveal novel insight into the virulence regulation in response to physiologically relevant signals.

### 3.3. Strain-Dependent Variations in SCFA Responses

Based on phylogenetic analyses, *L. monocytogenes* has multiple evolutionary lineages, with most isolates that cause human disease belonging to lineages I and II [52]. Alternatively, *L. monocytogenes* is also classified by serotyping somatic and flagellar antigens, in which serotypes 1/2a, 1/2b, 1/2c, and 4b are responsible for majority of the human listeriosis cases [53,54]. Variations in pathogenicity among isolates from different lineages and serotypes have been investigated and established through experimental infections as well as in vitro and in silico comparisons. For example, through in vitro hemolytic assays, it is clear that there exists a strain-dependent variation in the level of LLO production [55,56,57,58,59]. Additionally, as demonstrated through animal models of infections, there are strain-dependent variations in establishing infections in different organs [60,61]. These comparative studies are important for basic phylogenetic and phenotypic comparisons as well as epidemiological analyses that can help predict and prevent deadly outbreaks. 

To determine whether the ability to sense and respond to SCFAs is a relevant phenotype for common virulent strains of *L. monocytogenes*, we compared the effects of SCFAs between the lab strain 10403s, serotype 1/2a, and strain 07PF0776, a cardiotropic strain of *L. monocytogenes* belonging to serotype 4b [31,59]. Direct comparisons between these two strains have demonstrated that strain 07PF0776 established a significantly higher bacterial burden in the hearts of female ND4 Swiss Webster mice than strain 10403s after intravenous infection [59]. Using a rat cardiac myoblast cell line (H9c2) as a host model, although the intracellular growth curves were similar for the two stains, strain 07PF0776 exhibited a higher invasion rate than strain 10403s at 2 hours post infection [59]. Extending the comparisons to 9 additional strains revealed that the low versus high cardiac infections in mice correlated strongly with the internalin A (InlA) amino acid sequences [59]. In our study, while both strains are capable of altering LLO production in response to SCFAs, the direction of the effects were vastly different (Figure 4). The short-term effects of anaerobic SCFA exposure (Figure 5 and Figure 6) as well as long-term effects of SCFA exposure (Figure 7) on subsequent infections were also distinctively different between the strains. Because no SCFAs were included during infections, these strain-dependent variations are indicative of different adaptations to SCFAs prior to infections that ultimately affect subsequent intracellular success. While testing additional strains is needed to obtain a more complete lineage- or serotype-delineation of SCFA responses, it is possible that host physiology in the intestinal lumen may exert a selective force in *L. monocytogenes*-host co-evolution. 

### 3.4. Impact of SCFA Exposure on Subsequent Cell Culture Infections

Many enteric pathogens can sense and respond to SCFAs [62] although the mechanisms underlying SCFA responses vary from organism to organism. How these diverse SCFA responses manifest during aerobic versus anaerobic conditions is mostly unknown. While earlier work showed an enhancement of *L. monocytogenes* infections after exposure to anaerobic conditions [7,63,64], how SCFA exposure might influence the anaerobic enhancement of infections were unclear. Here, we used Caco-2 intestinal epithelial cell lines and RAW264.7 macrophages as infection models to study short term intracellular survival and invasion, respectively, and used L-cells to examine long term cell-cell spread—together to obtain a better understanding of the impact of SCFA exposure on the different stages of *L. monocytogenes* intracellular infections. It is important to note that because no SCFAs were included during infections so any SCFA treatment effects reflected bacterial adaptations and responses to SCFAs beyond the exposure period. 

Curiously, while anaerobic enhancement of invasion was a well-established phenomenon in *L. monocytogenes* infections [7,65,66], we did not observe similar behavior in our study (Figure 5). We believe this difference was caused by the utilization of 96-well tissue culture plates in this study compared to the 24-well tissue culture plates used in previous studies. It is likely that there were differences in oxygen diffusion rates naturally existing in these cell culture plates. In a 96-well plate, with a typical infection volume of 100 µL per well and a well diameter of 6.4 mm, there is an approximate 5 mm of liquid in height. In contrast, in a 24-well plate, with a typical infection volume of 500 µL and a well diameter of 15.6 mm, the liquid volume is approximately at 3 mm in height. Therefore, even though the multiplicity of infection was consistent, the infected cells were potentially exposed to different oxygen levels. If this variation in oxygen availability was the cause of the different infection outcomes, our results highlight yet another point during *L. monocytogenes*-host interactions where oxygen levels play an important role in influencing infection outcomes.

Strain 10403s and the isogenic mutants all showed similar trends in invasion and intracellular survival after aerobic or anaerobic exposure to SCFAs. Thus, regular PrfA and SigB activities likely do not play a role in facilitating responses to SCFA pre-treatments that are important for epithelial cell invasion or intracellular survival in macrophages. However, the isogenic mutants behaved very differently from the wildtype strain 10403s in the fibroblast infections (Figure 7). Prior exposure to M_lo_ concentration of SCFAs in the mutants resulted in significantly smaller plaque sizes compared to no SCFA pre-treatments. This indicates that PrfA and SigB play a role in *L. monocytogenes* adaptations to M_lo_ SCFAs that are relevant to success during prolonged infection. Additional studies are required to dissect the specific contributions each regulator might play in *L. monocytogenes* adaptations to SCFAs.

As the effects of SCFA treatments on infection outcomes did not align with in vitro effects on LLO production, it is likely that additional adaptations by *L. monocytogenes* play a strong role in survival and growth in host cells. LLO is vital for *L. monocytogenes* to escape the entry vesicles and grow within the cytosol. Thus, the level of LLO activity should be one determining factor in the success of intracellular *L. monocytogenes* at the early stage of the infection. At later stages of infections, intracellular *L. monocytogenes* was found to induce macrophage intracellular production of branched chain amino acids [65], which serve as the precursors for the synthesis of branched chain fatty acids in *L. monocytogenes* that could potentially counteract the effects of prior SCFA treatments. Whether SCFA-treated *L. monocytogenes* is equally efficient in stimulating macrophage branched chain amino acid production is unclear. Future studies on the signaling events by which macrophages respond to SCFA-adapted *L. monocytogenes* will provide more insight into the role of host response in the effects of SCFAs on host-pathogen interactions. 

### 3.5. Potential Implications for Transmission

The observed impact of SCFAs and oxygen levels on *L. monocytogenes* membrane fatty acid composition in this study, together with reported evidence of modifications in membrane fatty acid composition leading to compromised pathogenesis [8,27], support the importance of prior SCFA adaptations in the success of subsequent infections. Considering the spatial oxygen gradient *L. monocytogenes* likely experiences during transmission from the food matrices to the intestinal lumen and the intestinal epithelium, *L. monocytogenes* may undergo major membrane fatty acid modifications that can impact the initial host-pathogen interactions, leading to altered infection outcomes. Similarly, the presence of SCFAs, both as food preservatives [66,67,68] and as products of endogenous gut microbiota, may also alter *L. monocytogenes* membrane fatty acid composition to further influence subsequent infections. 

The active role of SCFAs in modulating *L. monocytogenes* membrane fatty acid composition highlights a potential explanation for individual variations in susceptibility to *L. monocytogenes* infections. It is commonly accepted that individuals such as the elderly and neonates are more susceptible to *L. monocytogenes* infections because of the weaker immune defenses. Curiously, the SCFA production likely declines during aging [69,70,71], a phenomenon suggesting that if a significant correlation between SCFA concentrations and susceptibility to *L. monocytogenes* infections existed, it might be possible to strengthen the defense in these high risk individuals against *L. monocytogenes* infections through non-invasive strategies such as an increase in dietary intake of fiber or probiotic organisms [72,73]. Moreover, the sustained, long-term effects of SCFAs on subsequent infections in the absence of SCFAs (Figure 5, Figure 6, Figure 7) suggest that the use of SCFAs in food storage may also be exploited to minimize the infection potential of *L. monocytogenes* upon accidental consumption. The ability of *L. monocytogenes* to sense and response to SCFAs provides a potential target to develop novel preventative or treatment strategies against *L. monocytogenes* at multiple stages of transmission.

In addition to membrane fatty acid modifications, the impact of SCFAs on LLO transcription and activity reported here also suggests potential effects on *L. monocytogenes* interactions with the intestinal epithelium by regulating LLO production. Several studies have shown that exogenously provided LLO is capable of activating mucin production and subsequent inhibition of *L. monocytogenes* internalization [74,75,76,77]. If exposures to SCFAs, likely in the anaerobic lumen of the intestines, could increase LLO production at the transcriptional level without immediate upregulation of the LLO activity (Figure 2), they may potentiate *L. monocytogenes* for subsequent intracellular infections, likely under conditions with higher levels of oxygen, without stimulating production of mucin that can negatively impact invasion. As *L. monocytogenes* moves toward the aerobic epithelium with reduced levels of SCFAs, the production and secretion of LLO resumes to promote infections. Extracellular LLO has been demonstrated to increase Ca^2+^ efflux in hepatocytes and promote subsequent internationalization of *L. monocytogenes* [78,79,80,81]. Therefore, the elevated level of LLO production upon aerobic exposure and the loss of inhibitory signals from SCFAs near the intestinal epithelium may promote *L. monocytogenes* invasion of the epithelial barrier. Future investigations into the activity of secreted LLO under physiologically relevant conditions in polarized epithelial cells may reveal novel insights on the intestinal phase of *L. monocytogenes* infections to better prevent *L. monocytogenes* from breaching the intestinal barrier and establishing lethal infections in peripheral organs.

## 4. Materials and Methods 

### 4.1. Strains

The *L. monocytogenes* strains used in this study include the wild-type strain 10403s and the isogenic mutants either containing a constitutively active PrfA transcriptional regulator [51] or a deletion in the gene encoding the stress response sigma factor SigB (Δ*sigB*) [82]. To quantify *hly* transcription, an *hly* transcriptional reporter strain (P*hly-gus-neo*, NF-L695, provided by Dr. Nancy Freitag at The University of Illinois College of Medicine) with the *hly* promoter driving the expression of β-glucuronidase (GUS) [83] was used. A cardiotropic strain 07PF0776 (provided by Dr. Nancy Freitag at The University of Illinois College of Medicine) [31] was also used in this study. Bacteria were streaked weekly on brain heart infusion (BHI) agar to provide fresh colonies for experiments. The *hly* reporter strain was grown on BHI agar containing neomycin.

### 4.2. Growth Conditions

*L. monocytogenes* cultures were grown overnight in filter-sterilized brain heart infusion media (BHI; Fisher Scientific #211059, Waltham, MA, USA) at 37 °C either aerobically with shaking at 250 rpm or anaerobically without shaking in an anaerobic chamber (Coy Laboratory, Type A, Grass Lake, MI, USA). The chamber contains a nitrogenous atmosphere and ~2% hydrogen. Sterilization of media was accomplished through filter sterilization to ensure consistency between batches. Buffered BHI was made with 100 mM sodium monobasic and dibasic phosphate.

### 4.3. Culture Supplementation

Separate stock solutions (1 M) of acetate (sodium acetate trihydrate; Fisher Scientific #121-09-3), propionate (propionic acid, sodium salt; Acros Organics #137-40-6), and butyrate (butyric acid, sodium salt; Acros Organics #156-54-7) were filter-sterilized and frozen in aliquots at −20 °C until needed. The salt forms of SCFAs were used to avoid acidification of culture pH by SCFA supplementations. Mixtures of SCFAs were prepared according to an earlier study [29] and included a low level of SCFA mixture (M_lo_: 25.5 mM acetate, 2.25 mM propionate, 2.25 mM butyrate) and a high level of SCFA mixture (M_hi_: 110 mM acetate, 70 mM propionate, 20 mM butyrate). These concentrations were chosen to ensure two different mixture levels were tested that fall within known ranges of SCFA levels [84,85]. 

### 4.4. GUS Reporter Assay

Cultures of the *hly* reporter strain were grown in BHI containing desired SCFA concentrations overnight at 37 °C aerobically or anaerobically. Bacteria from overnight cultures (1 mL) were harvested by centrifugation, washed in sterile PBS, and resuspended in sterile PBS (110 μL). Each sample was then sonicated for 30 s (kept on ice when not being sonicated) and centrifuged to remove cell debris and intact cells. A portion (10 μL) of the supernatant lysate was used to determine protein concentration by a BCA assay kit (Thermo Scientific #23227, Waltham, MA, USA) following the manufacturer’s protocol. The remaining lysate (100 μL) was placed into a 96-well flat bottom plate where 20 μL of a 4-methylumbelliferyl-β-D-glucuronide (Fisher Scientific #AAB21190MD) stock solution (0.35 mg/ml) was added in the dark. The plates were covered with foil and incubated at 37 °C for 10 min. Stop solution (10 μL of sodium carbonate at 0.2 M) was then added to the well in the dark and fluorescence (excitation 365 nm, emission 460 nm) was determined using a microplate reader (Biotek Synergy 4). 

### 4.5. Hemolytic Assay

Bacterial cultures were grown in 1 mL of BHI containing the desired SCFA concentration overnight at 37 °C aerobically or anaerobically. After measuring culture optical density at 600 nm, bacteria from overnight cultures (500 μL) were pelleted by centrifugation. Supernatant (100 μL) containing the secreted LLO was incubated at room temperature with 5 μL of 0.1 M dithiothreitol (DTT) for 15 min in a round bottom 96 well plate. The DTT-treated samples were serially diluted (1:2) with 100 μL of hemolytic assay buffer, containing 125 mM sodium chloride (Ameresco #0241-5KG, Framingham, MA, USA) and 35 mM sodium phosphate dibasic (Fisher 7558-79-4) at pH 5.5. Defibrinated sheep blood was prepared at 2% hematocrit in hemolytic assay buffer and was added to each well (100 μL per well), followed by incubation at 37 °C for 30 min. Plates were centrifuged at 2000 rpm for 5 min to pellet intact red blood cells and supernatant (120 μL) was transferred to a flat-bottom 96-well plate for absorbance reading at 541 nm using a microplate reader. Hemolytic unit was calculated by using the inverse dilution factor at half lysis normalized by OD.

### 4.6. FAME Analysis

Bacterial samples for fatty acid methyl esterification (FAME) analysis were harvested from overnight cultures (20 mL) grown at 37 °C by centrifugation at 10,000 rpm for 10 min. The pellet was then resuspended in 1 mL of sterile water and transferred to a microcentrifuge tube and spun again at 10,000 rpm for 3 min. The supernatant was removed, and the pellet was frozen in an ethanol-dry ice bath and sent to Microbial ID (Newark, DE, USA). The fatty acid composition was then determined using methyl esterification and gas chromatography.

### 4.7. Tissue Culture Infection

RAW264.7 macrophages and Caco-2 cells were cultured in Dulbecco’s Modified Eagle Medium (DMEM; Corning 10-013-CV, Corning, NY, USA) containing 10% FBS (Corning 35-010-CV) and penicillin/streptomycin (BioWhittaker 17-603E, Rockland, ME, USA) at 37 °C with 5% CO_2_. Cells were seeded into 96-well plates at 6 × 10^6^ cells per plate and incubated overnight. For infections, overnight *L. monocytogenes* cultures were centrifuged at 10,000 rpm for 3 min, washed once, and resuspended with sterile PBS. Culture OD was used to calculate the volume of bacterial culture suspension needed to achieve a multiplicity of infection of 10. After 30 min of infection in RAW264.7 and 1 h in Caco-2 cells, each well was washed twice with 1× DPBS (WorldWide Life Sciences, 61211088). DMEM with gentamicin (10 µg/mL) was added to each well to remove any extracellular *L. monocytogenes*. At 2 h post infection (hpi), adherent macrophages were lysed using filter-sterilized 0.1% Triton-X and the lysate was plated on LB plates. All LB plates were incubated at 37 °C for two days and colonies were counted using an aCOLyte 3 plate reader (Synbiosis). Percent input was calculated by dividing the number of intracellular colony forming units (CFU) at 2 hpi by the number of CFU in the inoculum and multiplying by 100%.

### 4.8. Fibroblast Plaque Assay

Murine fibroblast L-cells (ATCC CRL-2648) were grown in Dulbecco’s Modified Eagle Medium (DMEM) containing 10% Fetal Bovine Serum (FBS) in a flask. For experimentation, cells were seeded into six-well plates and given 24 h to form a monolayer. Overnight *L. monocytogenes* cultures were normalized by culture OD, washed, and resuspended in 100 µL of DPBS. Fibroblast monolayers were washed three times and infected with 1 mL of fresh DMEM containing 6 µL of *L. monocytogenes* suspension at no dilution, 1:10, and 1:100 dilution. At 1 h post infection, cells were covered with 3 mL of an overlay of DMEM containing 10 µg/mL gentamicin and 0.7% agarose. Plates were incubated at 37 °C with 5% CO_2_ incubator for 48 h. Following incubation, wells were stained with 1 mL of filter sterilized neutral red (0.33% (*w*/*v*) in DMEM) for one hour followed by three washes with DPBS. Plaques developed overnight in a 5% CO_2_ incubator and the plaque diameters were measured using the Gimp software (version 2.10.12).

### 4.9. Statistics

Statistical analyses include Student’s t-tests, which were performed using Microsoft Excel for pair-wise sample comparisons. Standard error of the mean (SEM) was used when the average of three independent experiments, each with three replicates, was calculated. Using SEM in these instances accounts for natural variation between experiments. Standard deviation (SD) was used when averages of three replicates from one independent experiment were used. 

## Figures and Tables

**Table 1:** Growth characteristics of *L. monocytogenes* strain 10403s in unbuffered brain heart infusion (BHI) with or without M_lo_ or M_hi_ short chain fatty acid (SCFA) mixtures. Asterisks, *, denote samples that showed a significant difference from the no supplementation control under the same oxygen levels where * *p* < 0.05, ** *p* < 0.01, *** *p* < 0.001. Averages of triplicates ± standard deviations are listed.

	SCFA	OD	pH	Doubling Time (Minutes)		
		WT	WT	WT	PrfA*	Δ*sigB*
Aerobic	No	0.96 ± 0.02	5.30 ± 0.02	74.73 ± 4.01	67.02 ± 1.49	74.02 ± 2.21
	M_hi_	0.90 ± 0.06	5.75 ± 0.01*	77.69 ± 4.11	70.01 ± 2.88	75.12 ± 3.34
	M_lo_	0.60 ± 0.01 *	6.16 ± 0.04 *	95.88 ± 5.18 *	96.00 ± 12.60 *	91.41 ± 4.93 **
Anaerobic	No	0.39 ± 0.02	4.78 ± 0.03	73.99 ± 2.03	80.39 ± 3.58	80.54 ± 2.28
	M_hi_	0.41 ± 0.01	5.20 ± 0.03 *	77.69 ± 3.51	83.17 ± 11.07	83.84 ± 2.43
	M_lo_	0.32 ± 0.03*	5.79 ± 0.07 *	108.87 ± 3.35 ***	119.61 ± 15.34 ***	126.59 ± 8.51 ***

**Table 2:** Overnight culture optical density (OD) and pH values in buffered BHI with or without M_lo_ or M_hi_ SCFA mixtures. Asterisks, *, denote samples that showed a significant difference from the no supplementation control under the same oxygen levels where * *p* < 0.05, ** *p* < 0.01, *** *p* < 0.001. NG represents cultures with no growth. Averages of triplicates ± standard deviations are listed.

	SCFA	Buffered BHI					
		6.0		7.0		8.0	
		OD	pH	OD	pH	OD	pH
Aerobic	no	0.87 ± 0.01	5.26 ± 0.03	0.74 ± 0.04	6.65 ± 0.02	0.38 ± 0.08	7.31 ± 0.01
	M_lo_	0.52 ± 0.07 **	5.67 ± 0.07 **	0.70 ± 0.03	6.73 ± 0.04	0.33 ± 0.07	7.32 ± 0.01
	M_hi_	NG	6.09 ± 0.0 ***	0.57 ± 0.0 **	6.72 ± 0.0 **	0.26 ± 0.05 **	7.36 ± 0.01 **
Anaerobic	no	0.19 ± 0.02	5.66 ± 0.02	0.40 ± 0.03	6.67 ± 0.01	0.11 ± 0.03	7.84 ± 0.02
	M_lo_	0.11 ± 0.03	5.78 ± 0.03 *	0.34 ± 0.03	6.67 ± 0.04	0.23 ± 0.02	7.90 ± 0.12
	M_hi_	NG	6.12 ± 0.03 ***	0.20 ± 0.2 **	6.68 ± 0.06	0.15 ± 0.01 **	7.55 ± 0.06 **

**Table 3:** Fatty acid composition (in percentages) in *L. monocytogenes* grown under aerobic conditions in BHI at 37 °C. Values represent averages of two independent replicates.

	Aerobic BHI		
	None	M_lo_	M_hi_
13 anteiso	0.06	0.07	0.07
15 anteiso	40.90	40.11	29.03
17 anteiso	36.26	30.82	11.31
19 anteiso	0.17	0.12	0
**Anteiso Total**	**77.39**	**71.11**	**40.41**
13 iso	0.06	0	0.09
15 iso	9.01	9.28	9.79
17 iso	3.93	3.58	2.31
**Odd Iso Total**	**12.99**	**12.86**	**12.18**
14 iso	0.48	0.83	2.11
16 iso	2.57	3.62	4.11
18 iso	0	0	0
**Even Iso Total**	**3.04**	**4.45**	**6.22**
**Branched Total**	**93.42**	**88.41**	**58.8**
11 straight	0	0	0.16
12 straight	0.11	0.17	0.65
13 straight	0	0.07	1.78
14 straight	0.30	1.18	5.45
16 straight	0	1.4	13.17
17 straight	4.54	7.21	15.67
18 straight	0	0.24	1.35
20 straight	1.72	1.25	2.87
**Straight Total**	**6.655**	**11.51**	**41.085**
16:1 w7c	0	0	0
18.1 w9c	0.08	0.08	0.11
**Unsaturated Total**	**0.08**	**0.08**	**0.11**
Branched to Straight Ratio	14.04	7.68	1.43
Culture OD	0.685 ± 0.10	0.74 ± 0.02	0.480 ± 0.01

**Table 4:** Fatty acid composition (in percentages) in *L. monocytogenes* grown under anaerobic conditions in BHI at 37 °C. Values represent averages of two independent replicates. DMA, dimethyl acetate; OD, optical density.

	Anaerobic BHI		
	No Supp	M_lo_	M_hi_
13 anteiso	0	0	0
15 anteiso	36.85	33.86	23.68
17 anteiso	24.86	21.06	8.12
19 anteiso	0.17	0.13	0
**Anteiso Total**	**61.88**	**55.05**	**31.80**
13 iso	0	0	0
15 iso	13.22	10.89	7.38
17 iso	5.25	4.32	1.91
**Odd Iso Total**	**18.47**	**15.21**	**9.29**
14 iso	1.27	1.41	1.53
16 iso	4.3	4.42	2.57
18 iso	0	0	0
**Even Iso Total**	**5.57**	**5.83**	**4.10**
**Branched Total**	**85.93**	**76.10**	**45.20**
11 straight	0	0	0.1
12 straight	0.19	0.27	0.35
13 straight	0	0.12	2.33
14 straight	0.56	2.23	5.08
15 Straight	0	2.815	25.39
16 straight	4.25	9.14	11.34
17 straight	0	0.41	2.11
18 straight	0.84	1.07	1.39
20 straight	0	0	0
**Straight Total**	**5.85**	**16.07**	**48.11**
16:1 w7c	0	0	0
18.1 w9c	0	0	0
**Unsaturated Total**	**0**	**0**	**0**
15:0 ISO aldehyde	0.29	0.2	0.1
unknown 13.493	0	0	0
14:0 2OH	0	0	0
14:0 DMA	0	0	0
15:0 iso DMA	1.04	0.71	0.31
15:0 DMA	0	0.21	1.89
16:0 aldehyde	0	0.19	0.30
UN 16.107	1.08	0.98	0.50
16:0 DMA	0.44	1.08	1.39
un 17.103	0.73	0.62	0.33
17:0 anteiso DMA	4.66	3.84	1.90
**Other Total**	**8.24**	**7.83**	**6.72**
Branched to Straight Ratio	14.67	4.73	0.94
Culture OD	0.524 ± 0.01	0.485 ± 0.00	0.301 ± 0.00
